# The Role and Efficacy of JNK Inhibition in Inducing Lung Cancer Cell Death Depend on the Concentration of Cisplatin

**DOI:** 10.1021/acsomega.4c01950

**Published:** 2024-06-18

**Authors:** Aurimas Stulpinas, Monika Tenkutytė, Aušra Imbrasaitė, Audronė V. Kalvelytė

**Affiliations:** Institute of Biochemistry, Life Sciences Center, Vilnius University, Vilnius 10257, Lithuania

## Abstract

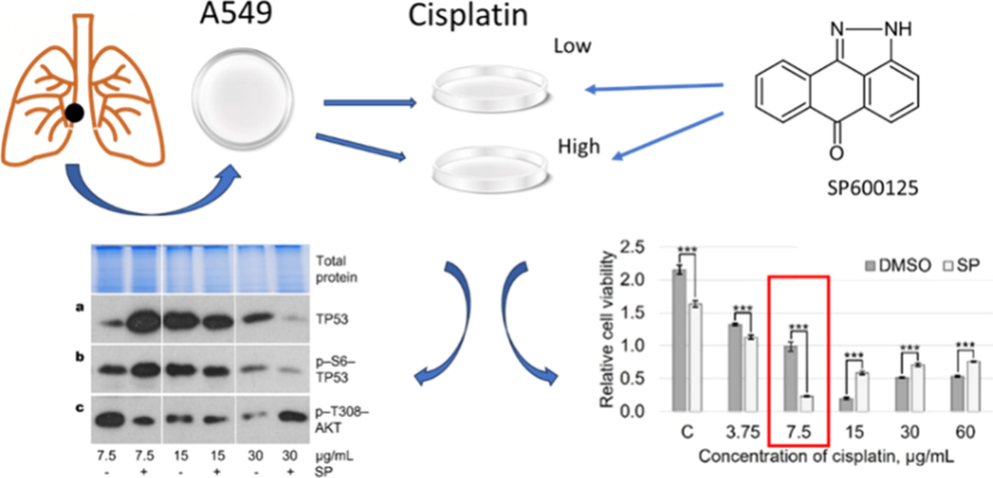

Toxicity and the emergence of resistance are the main challenges in cancer treatment. The optimal dose of cisplatin, one of the most widely used chemotherapeutic anticancer drugs, is currently being widely debated. Furthermore, the dose-dependent molecular mechanisms of its action are poorly understood. To assess the role of protein kinase JNK (cJun N-terminal kinase) signaling in lung cancer treatment, we combined small-molecule JNK inhibitors and cisplatin. Wild-type p53 (tumor suppressor transcription factor TP53) and mutated RAS-bearing lung adenocarcinoma cell line A549 was used as a model in our studies. Here, we demonstrate cisplatin concentration-dependent opposing roles of JNK in killing cancer cells: a cell-protective role at low cisplatin concentrations and an apoptosis-promoting (or neutral) role at high concentrations. Time- and dose-dependent activation of pro-survival protein kinase AKT and TP53 was shown, with similar activation dynamics in cells exposed to different (low and high) cisplatin concentrations. Selective inhibition of AKT and activation of TP53 (expression and phosphorylation) led to a decrease in cell survival, indicating their involvement in cisplatin-induced cell death regulation. The activation levels of TP53 and AKT in cisplatin-treated A549 cells after cotreatment with the JNK inhibitor SP600125 correlated with their role in regulating cell death. TP53 and AKT were proposed as signaling proteins mediating the outcome of JNK inhibition in A549 cells exposed to different concentrations of cisplatin. Our findings suggest that a combination of stress kinase JNK inhibition and low-dose cisplatin, together with manipulation of drug-induced signaling, could be considered as a promising treatment strategy for certain lung cancers.

## Introduction

The choice of therapy for the treatment of cancer represents a major challenge in defeating this disease. Various causes and mechanisms of resistance to treatment are known, the essence of which is the heterogeneity of tumor-forming cells, mainly determined by the plasticity of cancer cells, which in turn is controlled by various factors. In addition to genetic mutations, in most cases, nongenetic differences between the cells are responsible for this resistance. These include epigenetic changes, microenvironmental conditions, the presence of extrinsic growth-regulating factors, and cell-to-cell interactions, all of which ultimately result in altered signaling. It can be said that various external influences that change the state of the cell, and at the same time the intracellular signaling, can also change the sensitivity of the cells to treatment.

Advances in technologies and understanding of the signaling pathways lead to the discovery of new targets through which it is possible to improve treatment outcomes and patient compliance. At the same time, treatment methods have changed to a new trend, targeted therapy, a better treatment strategy with minimal side effects compared to chemotherapy. Unlike chemotherapy, targeted therapy affects tumor cells and usually causes less toxicity to healthy ones. Targeted therapeutics precisely aim at a specific molecular target that is found altered in a particular signaling pathway, and therefore, the cytotoxicity to healthy cells (a drawback of conventional chemotherapy) is avoided.

Various treatment options are currently available for non-small-cell lung cancer (NSCLC), like radiation therapy, chemotherapy, surgery, immunotherapy, and targeted therapy. In chemotherapy, various anticancer agents are used alone or in combination to treat the disease, among which are platinum-containing compounds and taxanes such as cisplatin and paclitaxel, respectively.

Cisplatin (cis-diamminedichloroplatinum II, cDDP) in combination with other cytotoxic agents is the most commonly used chemotherapy agent as a standard treatment regimen for a variety of solid tumors, including lung cancer. However, due to the accompanying toxic side effects as well as intrinsic, inherent, and acquired resistance of tumor cells, its effectiveness and, at the same time, its application are limited. Therefore, further studies of the molecular mechanisms of cisplatin action, including signaling pathways, and the search for new combinations of cisplatin with other regulators of cell functioning are needed as well as methods of their application and dependence on external influences. The rediscovery of platinum-based cancer therapy is a promising modern solution.^1,2^

The MAP kinase JNK (c-Jun N-terminal Kinase) is known as a stress response kinase and is often involved in inducing cell death. It is known to be involved in the regulation of key cell functions, such as proliferation and death. Dysregulation of the JNK signaling pathway can lead to neurodegenerative, cardiovascular, cancerous, and other diseases. JNK was first discovered as a kinase related to the oncogene c-Jun, i.e., phosphorylating this transcription factor (hence the name “c-Jun N-terminal kinase”, JNK), but JNK has also been shown to be activated by UV irradiation (hence the second name, stress kinase). Evidence suggests that the JNK pathway may be involved in human cancers. The first studies on stress kinases in lung cancer showed the activation of JNK in clinical samples. Other researchers have shown that JNK is activated in more than half of the non-small-cell cancer biopsy specimens studied and is involved in the malignant transformation of human bronchial epithelial cells.^3^ The increased phosphorylation of JNK in non-small-cell lung cancer compared to normal lung tissue suggests its potential importance in early-stage lung tumors and its role in bronchial epithelial oncogenesis.^4^ We hypothesized that JNK may be a differential diagnostic biomarker that distinguishes between two forms of non-small-cell lung cancer, adenocarcinoma or squamous cell carcinoma, which is important for the treatment of patients with Avastin. JNK is important in the differentiation and oncogenic transformation of squamous cell carcinoma cells^5,6^ and in the maintenance of cancer stem cell properties and stemness.^7^

Conversely, JNK1/2 may inhibit oncogenesis. It has been shown that JNK can activate an autophagy program to stop apoptosis or initiate a division program in cells adjacent to a dying cell.^8^ It has even been suggested that JNK1 is more involved in cell survival mechanisms as well as cancer promotion, while JNK2 is more involved in apoptosis and cancer inhibition.^9^ Moreover, JNK is actively involved in the emergence of drug resistance when treated with RAF (MAPK ERK signaling pathway)-targeted drugs (vemurafenib, dabrafenib, encorafenib), partly due to the fact that ERKs and JNKs share several phosphorylation targets, including c-Jun.^10^ Also, JNK pathway plays an important role in cisplatin drug resistance.^11^ Therefore, the oncogenic activity of JNK and its role in conferring cisplatin resistance suggest the inhibition of JNK as a strategy in cancer therapy.

This study was designed to elucidate the therapeutic potential of JNK inhibition in the treatment of lung cancer cells with cisplatin. Using the lung adenocarcinoma cell line A549 as a model, along with other patient-derived primary lung cancer cell lines, we demonstrated a cisplatin concentration-dependent, mutually/intrinsically antagonistic role of JNK in regulating cancer cell fate.

## Materials and Methods

### Chemicals

Cisplatin (Sigma-Aldrich, St. Luis, MO), dimethyl sulfoxide DMSO (99.5%, Sigma-Aldrich), resazurin (Sigma-Aldrich), MTT (Sigma-Aldrich), pifithrin-α (10 μM; Sigma-Aldrich), GSK-3beta inhibitor SB415286 (15 μM; Sigma-Aldrich), JNK inhibitor SP600125 (20 μM; Alfa Aesar, Ward Hill, MA), JNK inhibitor IX (2 μM; Merck, St. Luis, MO), nutlin-3a (10 μM; Merck), XG-102 (5 μM, in PBS; MedChemExpress, Monmouth Junction, NJ), bentamapimod (10 μM; MedChemExpress), AS601245 (10 μM; MedChemExpress), PI3K inhibitor Wortmannin (2 μM; Cayman Chemical, Ann Arbor, MI), capivasertib AZD5363 (10 μM; Cayman Chemical). Cell culture reagents: Iscove’s IMDM, CO_2_-independent medium, FBS, Glutamax 100× from Gibco (Grand Island, NY); antibiotic-antimycotic 100× and PBS 10× from Corning (Manassas, VA).

### Cell Culture

Human non-small-cell lung carcinoma A549 cells were purchased from Cell Lines Service GmbH, Eppelheim, Germany. Human primary lung cancer cell lines were established from surgical material (Regional bioethical approval no. 158200-18/5-1024-537) as previously described.^12^ Colorectal cancer cell line DLD-1 was obtained from the Proteomics Center of Life Sciences Center, Vilnius University (courtesy of Marija Ger). All cells were cultivated in a humidified incubator at 37 °C and 5% CO_2_ in IMDM (Gibco) with 10% FBS (Gibco) and 1× antibiotic-antimycotic solution (Corning). The cell cultures were periodically treated with Biomyc-3 (Ciprofloxacin) according to the manufacturer’s (Biological Industries, Kibbutz Beit Haemek, Israel) instructions to prevent contamination with mycoplasma.

### Cell Viability

**Figure 1:**
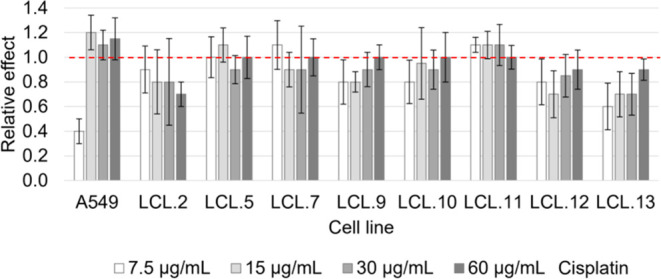
Effect of the MAP kinase JNK inhibitor SP600125 (20 μM) on the viability of cisplatin-treated lung cancer line cells. Different concentrations of cisplatin were used. The ratio of the MTT value of samples containing cisplatin + SP600125 to the MTT value of samples treated with cisplatin + DMSO alone of different cell lines is shown. Cell viability was determined after 72 h of treatment. “Relative effect” value of 1.0 (a dashed line) means that SP600125 had no effect on cell survival. LCL indicates the primary cell lines used. Concentrations of cisplatin are indicated in the figure. Data are expressed as mean ± SD, *N* = 5.

Cell viability after treatment was measured using the MTT assay. Briefly, cells were seeded into cell culture-treated 96-well plates on day −1 to reach confluence the next day. On day 0, no less than four wells were tested for their viability using MTT (3-(4,5-dimethylthiazolyl-2)-2,5-diphenyltetrazolium bromide from Sigma-Aldrich) reduction assay (1 h, 0.2 mg/mL of MTT in PBS). After removal of the MTT solution from the initial control, other wells were subjected to cisplatin and combination treatments in fresh IMDM medium with 10% FBS and 1% antibiotics. On day 3 (72 h of treatment), MTT solution was aspirated and discarded. Water-insoluble MTT formazan was dissolved in ethanol and quantified by spectrophotometrically reading absorbance at 570 nm in a Varioskan Flash plate reader. “Relative viability” in the figures refers to the ratio of the measured values after the treatment to the baseline control (in cisplatin dose−response experiments). “Relative effect” in Figure 1 refers to the ratio of the MTT values of samples containing cisplatin + SP600125 to the MTT values of samples treated with cisplatin + DMSO alone. A magnitude of 1.0 means that SP600125 had no effect.

Alternatively, for extracellular contact studies, a resazurin (7-hydroxy-3H-phenoxazin-3-one-10-oxide sodium salt) reduction assay was used to evaluate the cell viability. Briefly, a solution of resazurin (0.5 mg/mL stock solution dissolved in PBS) is added to the growth medium with cells (both adherent and in suspension) to make a 20 μg/mL final concentration. The plate is incubated for 2 h at a temperature of 37 °C. Fluorescence of the reduced form of resazurin 545/590 nm is recorded with a plate reader.

### Apoptosis Assay

The mode and percentage of cell death were determined by fluorescence microscopy using a mixture of two fluorescent dyes: Acridine orange and Ethidium bromide (AO/EB, from Sigma-Aldrich), with the final concentration of 100 μg/mL of each. AO dye was used to visualize chromatin condensation while EB discriminated cell membrane integrity in order to identify the apoptotic cell morphology and fraction in the population as described by Mercille and Massie.^13^

### Agitation Experiments

To mimic the anchorage-independent state of circulating cells, we maintained the cells in suspension for 24−72 h during the experiments. To prevent their adhesion to tissue culture plates, trypsin-detached cells in a CO_2_-independent medium (supplemented with 10% FBS, 2 mM glutamine, 1× antibiotics) were placed in nontreated cell culture flasks (Eppendorf Austria GmbH, Vienna, Austria) and constantly agitated at 90 rpm (orbit 20 mm) for 24 h in an environmental orbital shaker-incubator. For further experiments, the suspension was concentrated by centrifugation, resuspended in fresh medium, and transferred into nontreated 24-well plates (Eppendorf) where cisplatin and inhibitors were added for cytotoxicity studies.

### Western Blotting

For the protein expression and phosphorylation analysis, cells were lysed in lysis buffer (10 mM Tris-HCl (pH 7.4), 50 mM NaCl, 5 mM EDTA, 50 mM NaF, 1% Triton X-100, 1 mM PMSF, 2 mM Na_3_VO_4_, 20 μg/mL aprotinin). An equal amount of protein (Bradford assay; Sigma-Aldrich) was run in SDS-PAGE. Proteins were transferred onto a PVDF membrane using semidry transfer. After blocking with 5% nonfat milk powder in TBST, antiphospho-protein antibodies of selected signaling molecules were used (4 °C, overnight) before visualizing with secondary HRP-conjugated antibodies (goat antirabbit and goat antimouse) from Bio-Rad Laboratories, Inc. (Hercules, CA) and an enhanced chemiluminescence reagent (Bio-Rad). Western blot images were obtained by exposing the membranes to X-ray film (Carestream Health, Rochester, NY) for several different time periods in order to visualize the signals of different intensities, avoiding overexposition. The films were further developed according to the manufacturer’s instructions and scanned at 400−600 dpi resolution. In many cases, the same blotting membrane has been reprobed with other antibodies. Representative Western blots from at least 3 independent experiments that resulted in the same outcomes are presented. A part of the gel with 100+ kDa mass proteins was stained with Coomassie R-250 brilliant blue dye (Thermo Scientific) to serve as loading controls, as suggested instead of housekeeping proteins.^14,15^

Primary antibodies used: pT308 Akt (Cell Signaling Technology, #2965), AKT (Invitrogen, 44-609G), pGSK3beta (Cell Signaling, #5558), pT183/pY185 JNK (BD, #612540), c-Jun (BD, #610326), pS63 c-Jun (BD, #558036), JNK D-2 (Santa Cruz, #sc-7345), p53 (Santa Cruz, sc-6243), pS6 p53 (Abcam, 32132).

### Statistical Analysis

The data in the charts are expressed as means (±SD) of at least five independent experiments performed in quadruplicate. Paired *t*-test to compare two means was used for statistical analysis (GraphPad Software, Inc., La Jolla, CA). Differences were considered statistically significant (*) at *p* < 0.05, (**) *p* < 0.005, (***) *p* < 0.0005. The cytotoxicity experiments were performed in quadruplicate for each time point and each concentration, and repeated more than three times.

## Results

The main studies were performed with A549 cells, the non-small-cell lung carcinoma, known as a wild-type tumor suppressor TP53-bearing and KRAS G12S mutation-driven cancer cell line. In addition, a panel of genotypically and phenotypically different patient-derived lung cancer (NSCLC) primary cell lines was used in our studies. Cell lines were obtained from patients without genetic characterization (Regional bioethical approval no. 158200-18/5-1024-537).

Our earlier studies revealed that our established human non-small-cell lung cancer-derived primary cell lines variously expressed putative lung cancer stem cell surface markers, showed cells of different morphology, epithelial or mesenchymal phenotypes, which were differently positive for stemness- and EMT-related transcription factors.^12,16^

Current studies using the JNK inhibitor SP600125 showed that the role of JNK in the lung cancer cell lines studied and in adenocarcinoma A549 cells is different (Figure 1). In A549 cells, the role of JNK depends on the concentration of cisplatin and can change from antiapoptotic to pro-apoptotic or neutral. At a low concentration of cisplatin, 7.5 μg/mL (25 μM), JNK protects cells from death, its inhibition effectively induces apoptotic cell death. This effect is observed when cells are grown in the presence of serum or in a serum-free culture medium without growth factors (Supplement Figure S1).

**Figure 2:**
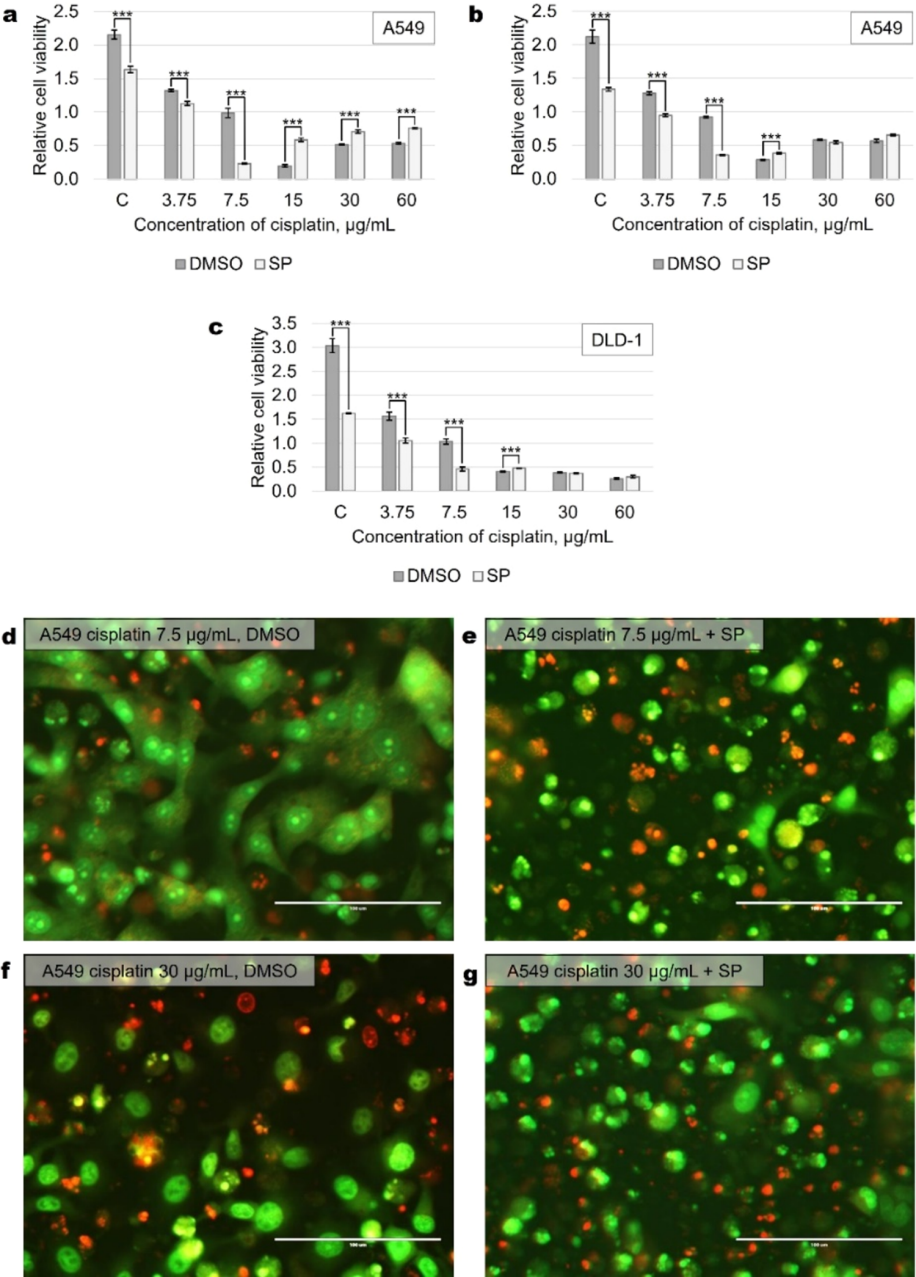
Effect of JNK inhibition on A549 cell viability as a function of cisplatin concentration. (a, b) 7.5 μg/mL concentration of cisplatin becomes lethal in combination with JNK inhibitor SP600125. However, at higher cisplatin concentrations, the inhibitor either protects against cisplatin-induced cell death (a) or does not have any effect (b). (c) Similar effects are observed in the colon cancer cell line DLD-1. (d–g) SP600125 potentiates the appearance of cells with apoptotic morphology in 7.5 μg/mL cisplatin-treated A549 cells (d, e), in contrast to 30 μg/mL cisplatin-treated A549 cells (f, g). Relative cell viability is cell viability after 72 h treatment normalized by initial viability (measured by the MTT method). Representative test results (all measurements were performed in quadruplicate) from more than five experiments are presented. Fluorescent pictures were obtained with a mixture of AO/EB dyes as described in the Methods section. SP—JNK inhibitor SP600125 (20 μM).

Thus, we further investigated the role of the combination of the JNK inhibitor SP600125 with cisplatin in A549 cells. Our study used the ATP-competitive anthrapyrazole inhibitor SP600125 to determine the involvement of JNK in lung cancer cell death regulation during cisplatin treatment. JNK was found to be required for the proliferation of the cells studied. A dual role of JNK was shown in A549 cells during cisplatin treatment. The studies revealed that JNK plays a cell-protective role at low cisplatin concentrations but an apoptosis-promoting or neutral role at high cisplatin concentrations. In Figure 2, presented data show that at 7.5 μg/mL cisplatin concentration, JNK inhibitor SP600125 effectively enhanced cell death, while increasing the cisplatin concentration 2-fold or more resulted in cell-protective or neutral result (Figure 2a,b).

It is known that A549 is a *RAS-*mutated cell line. The same as in A549 dependence of the role of JNK on cisplatin concentration was found with another cancer cell line, colon cancer DLD-1 cells harboring KRAS mutations, among other mutations (Figure 2c).

However, such variation in the role of JNK at different concentrations of cisplatin was not detected in primary cell lines with an unknown mutational profile (Figure 1). Since primary cell line cells did not exhibit the effect of JNK inhibition on cell viability at low cisplatin concentration found in A549 cells, we can presume that the cisplatin concentration-dependent change in the role of JNK is not specific to lung cancer cells; it rather depends on the oncogenic mutation. The mode of cell death induced by the cisplatin and SP600125 combination is apoptosis (Figure 2d−g).

Therefore, we found that JNK inhibition at low cisplatin concentrations is highly effective at killing non-small-cell lung cancer A549 cells. Since both A549 and DLD-1 cell lines harbor activating *KRAS* mutations (Cellosaurus.org, 2023), we hypothesize that the difference in JNK functions at different concentrations of cisplatin may be due to increased KRAS activity.

**Figure 3:**
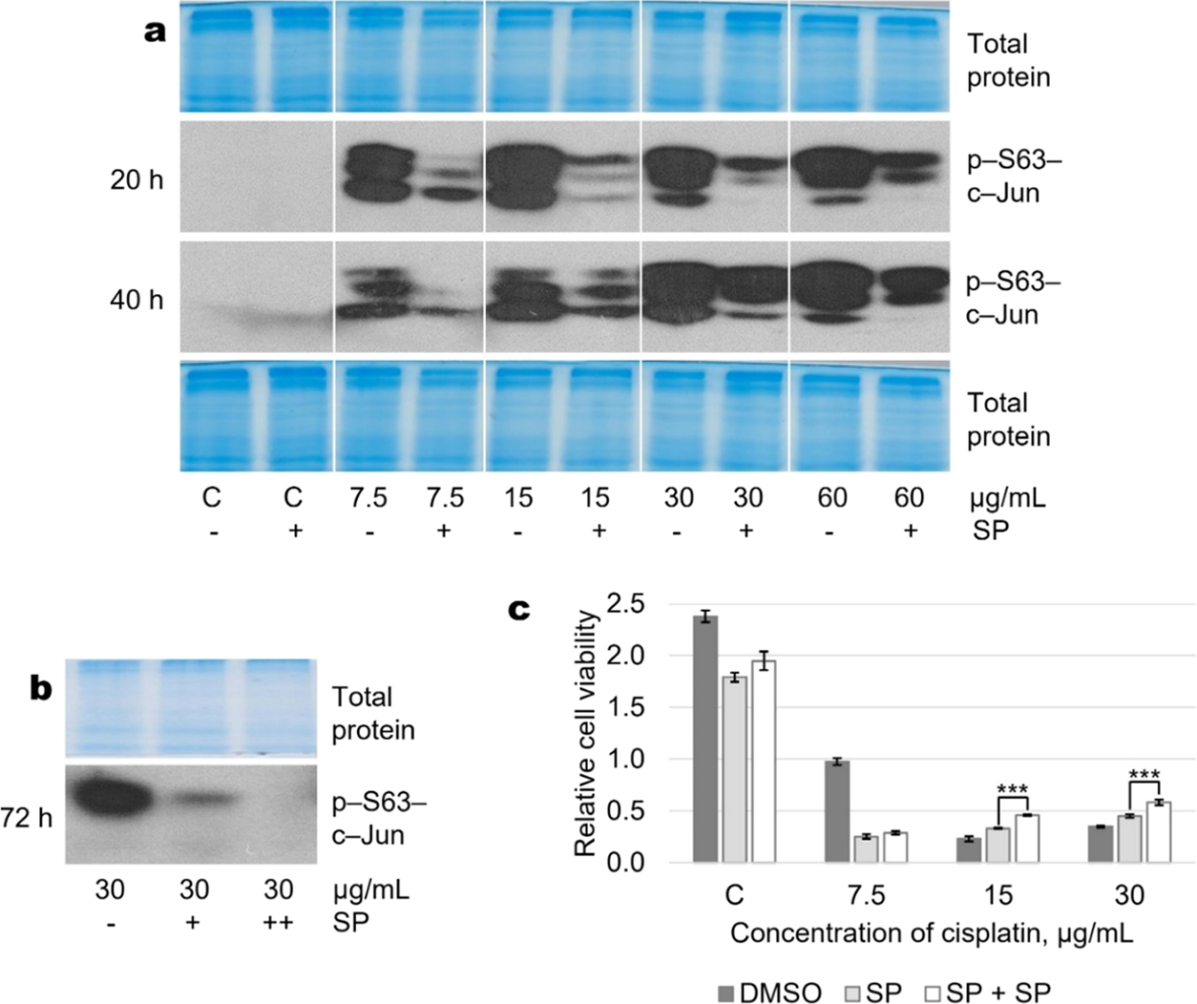
JNK inhibitor SP600125 reduces JNK target transcription factor c-Jun phosphorylation in cisplatin-treated A549 cells. Different concentrations of cisplatin were used. (a) Representative Western blots from 20 and 40 h of treatments are shown. Total protein Coomassie-stained polyacrylamide gels serve as loading controls. (b) Repeated addition (after 24 h) of SP potentiates suppression of c-Jun phosphorylation in response to cisplatin treatment. (c) Repeated addition of SP strengthens the protective effect of JNK inhibition at 15−30 μg/mL concentrations of cisplatin in A549 cells. SP—JNK inhibitor SP600125 (20 μM). p*** < 0.0005, *N* = 3.

Further studies confirmed effective inhibition of JNK target c-Jun phosphorylation after SP600125 exposure at all concentrations of cisplatin, although recovery of c-Jun phosphorylation was seen at higher cisplatin concentrations as treatment time increased (40 h) (Figure 3a). Repeated addition of the JNK inhibitor SP600125 inhibited this restoration of c-Jun phosphorylation at high concentrations of cisplatin (Figure 3b), but it did not alter the role of JNK in cell survival (Figure 3c). We thus confirmed the cisplatin concentration-dependent differential role of JNK in A549 cells.

**Figure 4:**
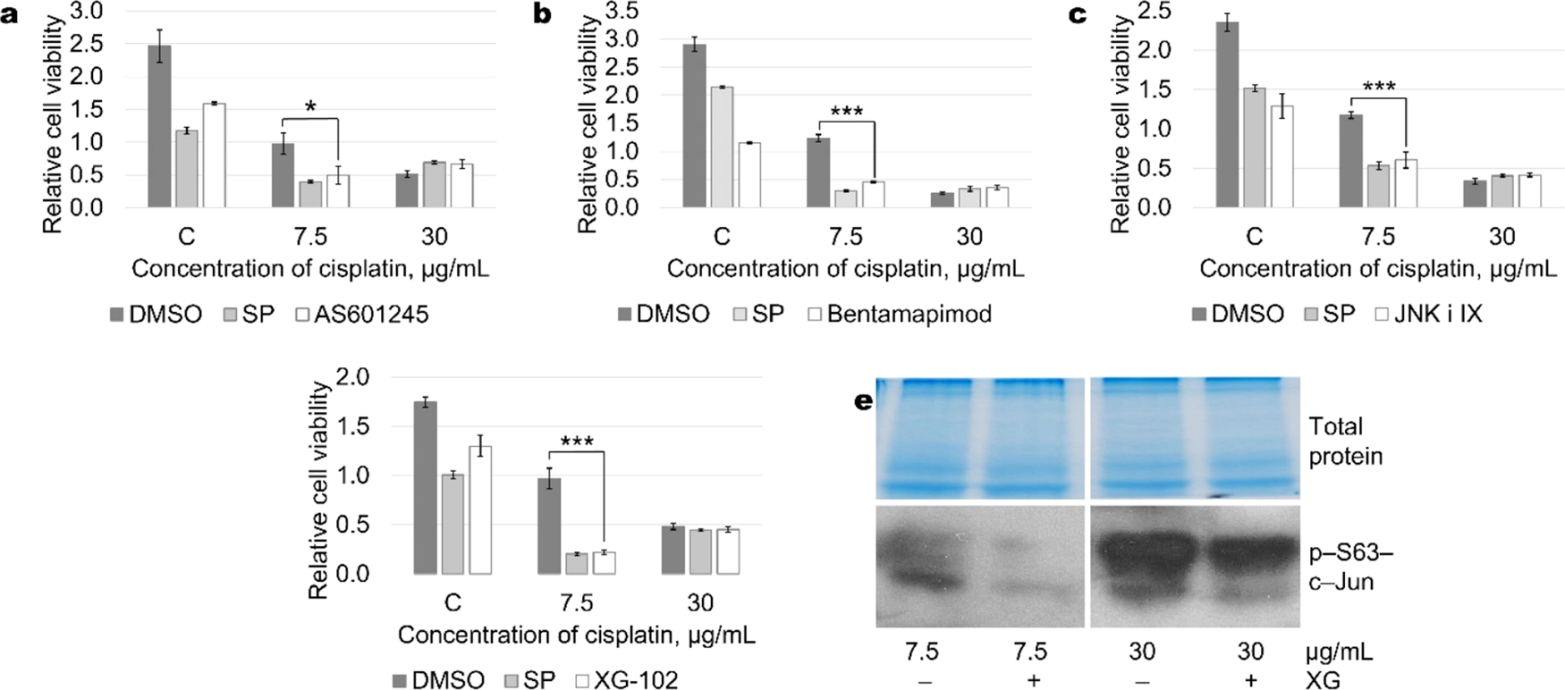
Cisplatin concentration-dependent effect of different JNK inhibitors on A549 cell viability. Different JNK inhibitors show the same dependence on cisplatin concentration on cell viability. Statistically significant reduction in cell viability is observed at 7.5 μg/mL cisplatin in combination with 10 μM AS601245 (a), 10 μM bentamapimod (b), 2 μM JNK inhibitor IX (c), and 5 μM XG-102 (d). Representative test results (all measurements were performed in quadruplicate) from more than five experiments are presented. (e) Reduction in c-Jun phosphorylation upon the addition of JNK inhibitor XG-102, as determined by Western blot. Representative Western blots are shown. Total protein Coomassie-stained polyacrylamide gels serve as loading controls.

To confirm the role of JNK signaling dependence on the cisplatin concentration in cell viability rather than the unspecific effects of the inhibitor SP600125, we further evaluated the efficacy of other JNK inhibitors in A549 cells treated with different concentrations of cisplatin. We performed analogous experiments using different JNK inhibitors: ATP-competitive inhibitors AS601245, bentamapimod, JNK2 inhibitor IX (JNK-i-IX), and a peptide-based inhibitor XG-102, acting outside of ATP binding pocket. As shown in Figure 4, the aforementioned inhibitors of JNK in combination with cisplatin exhibited the same cisplatin dose-dependent effect as previously demonstrated with SP600125. A significant reduction in cell viability was observed at a low (7.5 μg/mL) cisplatin concentration after exposure to all tested JNK inhibitors regardless of their mechanism of action. Therefore, we can state that the inhibition of the JNK pathway determines the fate of the cell independently of the inhibitor used.

**Figure 5:**
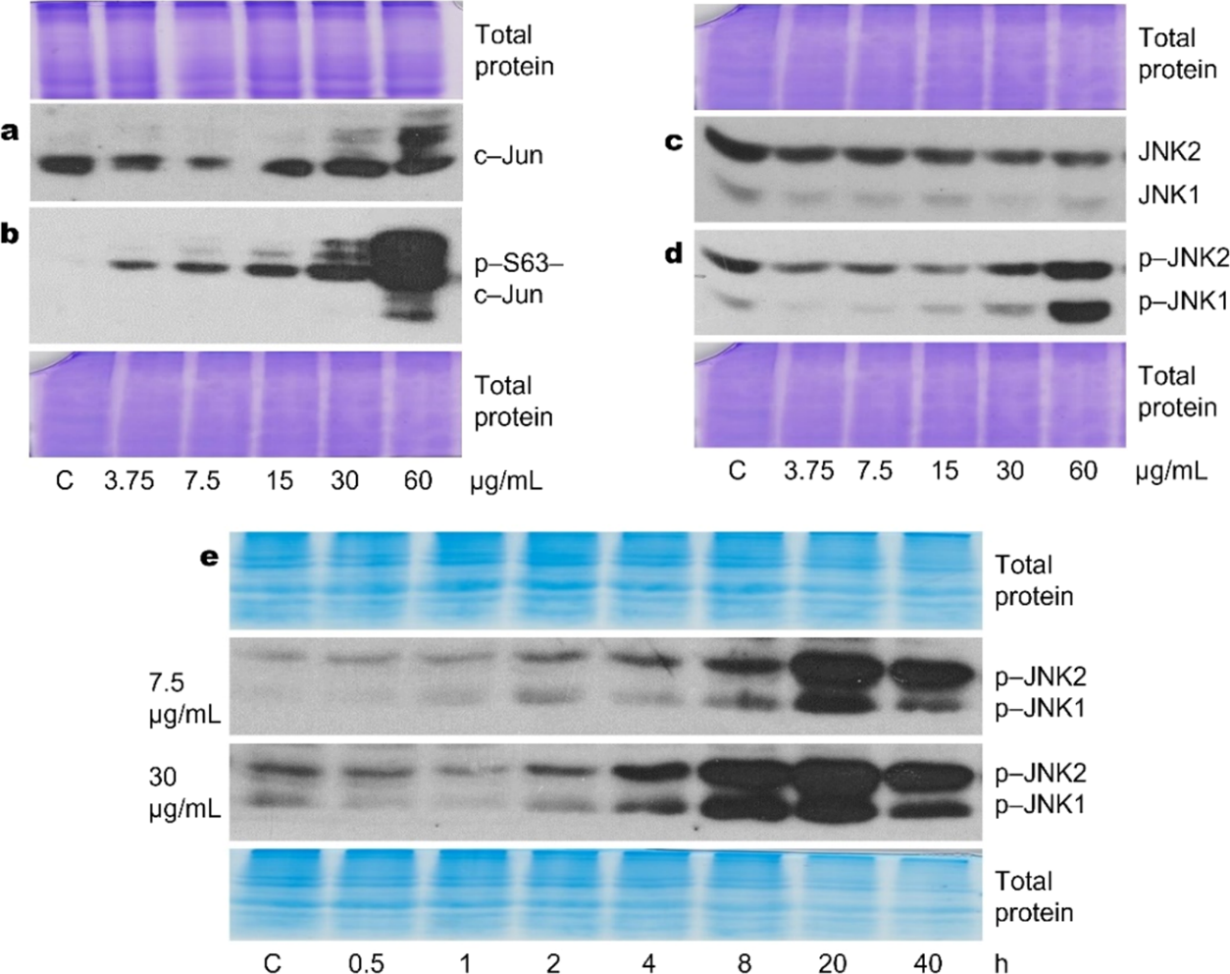
Expression and activation of JNK signaling pathway molecules induced by cisplatin. (a, b) c-Jun protein expression and phosphorylation (S63) increase with increasing cisplatin concentration. (c) Cisplatin does not affect JNK1/2 protein expression. (d) JNK phosphorylation increases with increasing cisplatin concentration. Western blots show the JNK phosphorylation status following 6 h of treatment. (e) Two different cisplatin concentrations result in the same gradual long-term activation of JNK. Representative Western blots are shown. Total protein Coomassie-stained polyacrylamide gels serve as loading controls.

It is known that different dynamics of protein activation in a cell can determine whether it will survive or die, so we investigated whether the different effect of JNK inhibition in A549 cells is due to different kinetics of JNK activation. As is widely accepted, cell survival should be potentiated by its transient, short-term activation. Researching the signaling pathways and expression of signaling molecules induced by cisplatin, we showed that JNK phosphorylation, JNK target c-Jun amount, and phosphorylation increased with increasing cisplatin concentration. Cisplatin did not affect the content of total JNK in the cells (Figure 5a−d).

Gradual and prolonged increase of JNK and its target transcription factor c-Jun phosphorylation was observed in A549 (Figure 5) and other lung cancer-derived cell lines (data not presented) after cisplatin treatment. Meanwhile, the expression of these signaling molecules differed: JNK expression did not change while c-Jun expression increased during cisplatin treatment (Figure 5a,c). We showed that there was no apparent difference in the kinetics of JNK activation at both tested concentrations of cisplatin (7.5 and 30 μg/mL; Figure 5e). Thus, JNK-inhibition-mediated death or survival is not determined by the differences in the kinetics of JNK activation.

The mechanism of action of cisplatin involves the activation of multiple signaling pathways, leading to cell death. To elucidate potential targets of JNK pathway inhibition, next, we investigated the activation of transcription factor TP53 and the survival kinase AKT in cisplatin-treated A549 cells.

**Figure 6:**
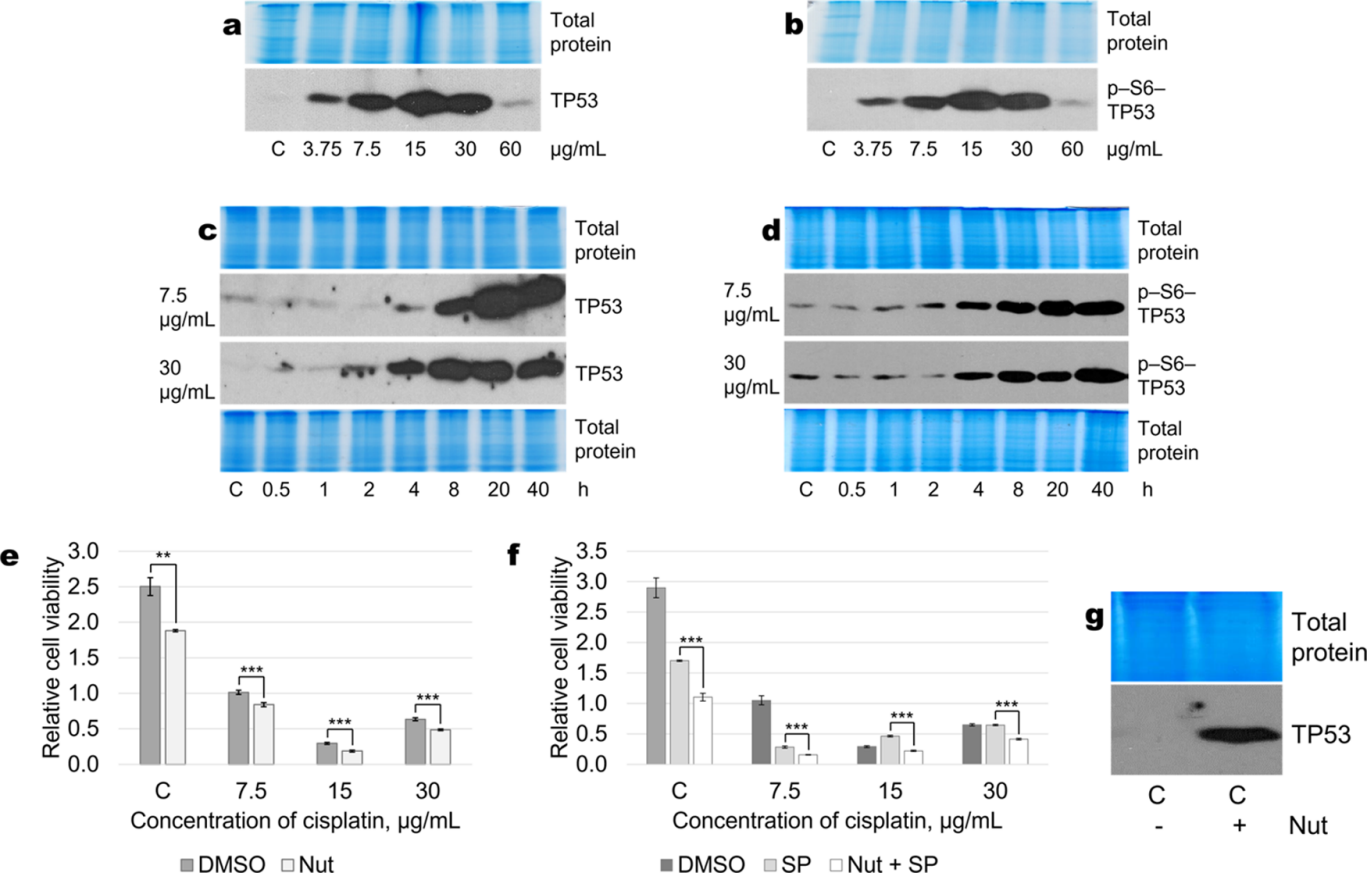
Activation of TP53 in A549 cells in response to different concentrations of cisplatin. (a, b) Expression and phosphorylation at serine-6 is induced by cisplatin and is maximal at 15 μg/mL (6 h of cisplatin treatment). (c, d) Prolonged and increasing expression and phosphorylation of TP53 in cells treated with either 7.5 μg/mL or 30 μg/mL of cisplatin. (e) TP53 activator nutlin-3a potentiates cisplatin-induced decrease in cell viability. (f) Nutlin-3a reduces viability of cisplatin + SP600125-treated cells. C—control without cisplatin; Nut—nutlin-3a (10 μM); SP—SP600125 (20 μM). Representative test results (all measurements were performed in quadruplicate) from more than five experiments are presented, p** < 0.005, p*** < 0.0005, *N* = 4. (g) Nutlin induces expression of TP53. Representative Western blots are shown. Total protein Coomassie-stained polyacrylamide gels serve as loading controls.

By studying the expression, phosphorylation, and kinetics of TP53 activation after exposure to different concentrations of cisplatin, we found that depending on cisplatin concentration, the amount and phosphorylation of the tumor suppressor TP53 increased (Figure 6a,b). To determine whether the dynamics of TP53 activation differ between cells exposed to different concentrations of cisplatin, we treated them with 7.5 or 30 μg/mL cisplatin for 0.5−40 h. The amount and phosphorylation of TP53 in the cells increased with increasing the duration of exposure. However, the activation kinetics of this protein did not differ between different concentrations (Figure 6c,d).

Next, we sought to elucidate the role of identified TP53 activation in regulating cell death after exposure to cisplatin. In this study, pifithrin-α (PFT-α) was used to inhibit p53 functioning. PFT-α is known as a specific p53 inhibitor that selectively blocks the transcriptional activity of the tumor suppressor p53 and is commonly used to distinguish between p53-dependent and -independent apoptosis as well as to prevent severe side effects often associated with chemotherapy and radiotherapy. However, current evidence suggests that PFT-α only partially inhibits p53 function and protects cells from DNA damage-induced apoptosis also by p53-independent mechanisms. Unfortunately, our studies did not show an unequivocal answer regarding the role of PFT-α in regulating the death of our studied cells, both exposed to cisplatin alone and in combination with the JNK inhibitor SP. Therefore, the p53 agonist nutlin-3a was chosen as a regulator of p53 activity instead. Nutlin-3a is known to inhibit the interaction between MDM2 and TP53, thereby stabilizing and increasing the p53 levels in wild-type p53 cells. Upon treatment of A549 cells with nutlin, we observed an inhibition of cell proliferation and an increase in cell death at different cisplatin concentrations both alone (cisplatin + nutlin Figure 6e) and in combination with JNK inhibitor SP600125 (cisplatin + SP + nutlin; Figure 6f), suggesting an increase in the pro-apoptotic function of TP53. Activation of TP53 expression by nutlin is shown in Figure 6g.

Therefore, we found that similar TP53 dynamics were generated in response to the different cisplatin concentrations, which, as described earlier, lead to different cell fate outcomes. Consequently, the p53 dynamics alone was insufficient to determine the cause of opposite cell fates in response to different doses of cisplatin. Therefore, other signaling pathways operating in parallel may be responsible for the generation of different responses.

**Figure 7:**
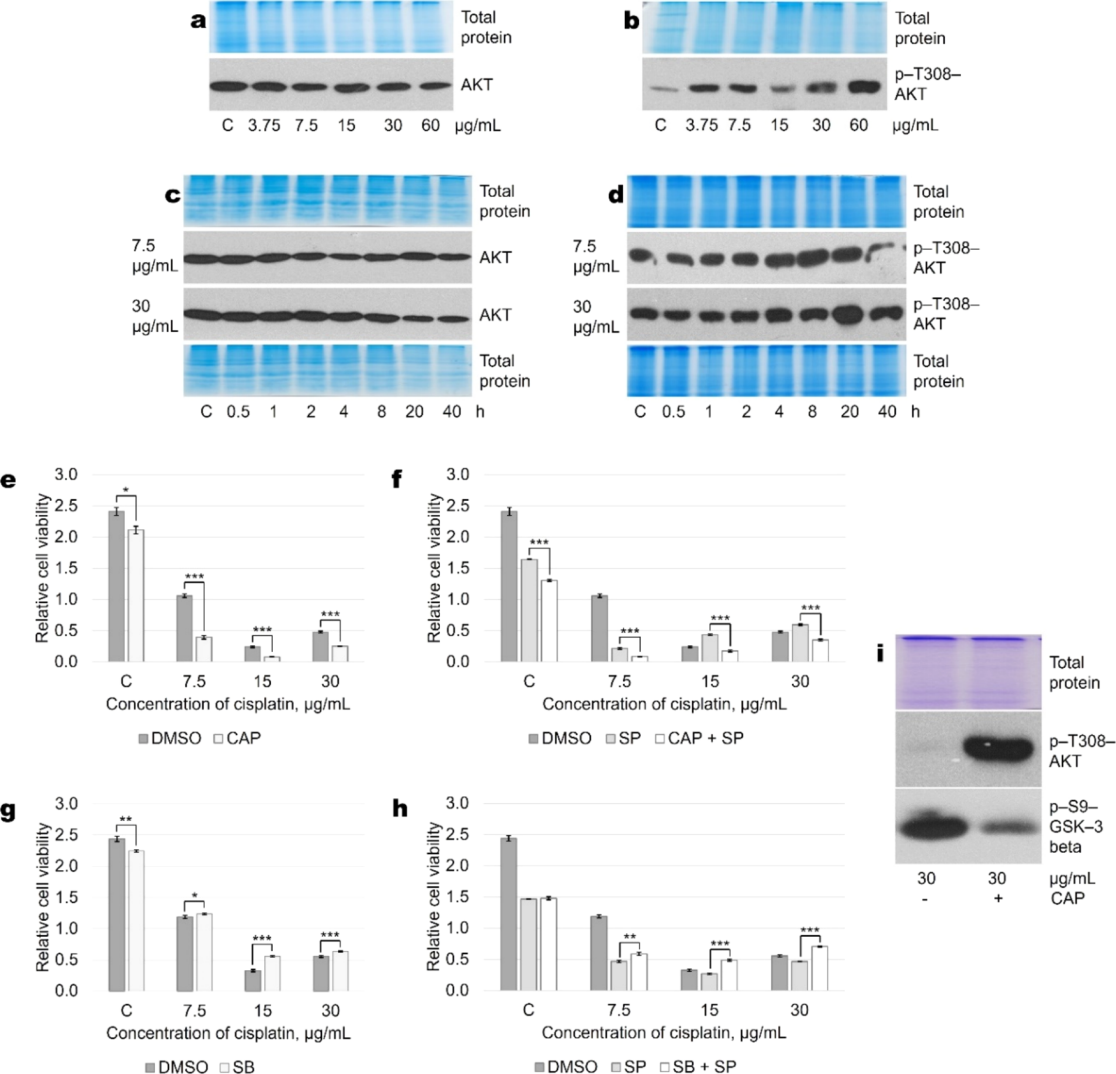
Involvement of AKT signaling pathway in cisplatin-induced A549 cell death. (a) AKT protein level does not depend on cisplatin concentration. 6-h-long exposure is presented in the Western blot picture. (b) Phosphorylation/activation of AKT is cisplatin concentration-dependent. 6-h-long exposure is presented in the Western blot picture. (c) AKT protein level does not change during 40 h of cisplatin treatment. (d) Dynamics of AKT activation following cisplatin (7.5 or 30 μg/mL) treatment. (e, f) AKT inhibitor capivasertib enhances cell death at all concentrations of cisplatin used both in the absence (e) and presence (f) of JNK inhibitor SP600125. (g, h) GSK3-β inhibitor SB415286 protects A549 cells from cisplatin both in the absence (g) and presence (h) of JNK inhibition. Representative test results (all measurements were performed in quadruplicate) from more than five experiments are presented. p*<0.05, p**<0.005, p***<0.0005, *N* = 4. (i) Capivasertib inhibits AKT activity as shown by the inhibition of AKT molecular target GSK3-β phosphorylation at serine-9. Representative Western blots are shown. Total protein Coomassie-stained polyacrylamide gels serve as loading controls. C—control without cisplatin; CAP—capivasertib (10 μM); SP—SP600125 (20 μM), SB—GSK3 inhibitor SB415286 (15 μM).

In search of other possible participants in the action of cisplatin, we found that treatment of cells with cisplatin increased the activation of the pro-survival AKT kinase. Dynamics of the activating AKT phosphorylation may also be responsible for the observed differences in cell fate determination caused by low and high cisplatin concentrations. Hence, by using Western blot analysis to detect total (Figure 7a) and phosphorylated forms of the kinase (Figure 7b) we showed that activation of the AKT was cisplatin dose-dependent with a maximum kinase activation at 60 μg/mL. To determine whether activation dynamics of AKT differ between A549 cells exposed to different drug concentrations, we treated cells with 7.5 and 30 μg/mL of cisplatin. As shown in Figure 7, the total protein level of AKT was stable (Figure 7c), neither the concentration of cisplatin nor the duration of exposure affected the amount of total AKT protein in the cells. However, the phosphorylation of AKT mounted as cisplatin concentration increased, and the duration of exposure increased (Figure 7d). Thus, while the combination of different cisplatin concentrations and JNK inhibition induced different cell fate outcomes in A549 cells, both cisplatin doses resulted in similar AKT phosphorylation dynamics.

Therefore, it can be stated that in A549 cells, AKT phosphorylation dynamics also does not determine cisplatin concentration-dependent cell death or survival induced by JNK inhibitors. Furthermore, by using the AKT inhibitor capivasertib, we found that at all tested concentrations of cisplatin, AKT inhibition increased cell death, i.e., AKT performed a protective (antiapoptotic) function in the cells (Figure 7e). We also demonstrate that capivasertib in combination with SP and cisplatin increased A549 cell death compared with cells exposed to cisplatin + SP alone (Figure 7f).

To see if there may be a signaling mediator downstream of AKT responsible for the aforementioned phenomenon, we investigated the role of glycogen synthase kinase-3 β (GSK-3β) in cisplatin-induced A549 cell death. GSK-3β is known to be phosphorylated and inactivated by the phosphoinositide 3 kinase (PI3K/AKT) pathway.^17^ GSK-3β has been reported to promote cell growth and survival in colon, breast, ovarian, and pancreatic adenocarcinomas. Therefore, we used a specific GSK3 inhibitor SB216763 and found that SB protected lung adenocarcinoma A549 cells from cisplatin in both the absence (Figure 7g) and presence (Figure 7h) of JNK inhibition. These data go in parallel with capivasertib inhibition of AKT, as confirmed by Western blot analysis: although treatment with CAP increased the phosphorylation of Akt itself, in agreement with our previous studies,^16^ it prevented phosphorylation of AKT target GSK-3 β (Figure 7i). Similar results regarding cell viability were obtained with another inhibitor of the AKT pathway, phosphatidylinositol 3-kinase (PI3K) inhibitor Wortmannin (Supplement Figure S2).

Thus, in A549 cells, TP53 is pro-apoptotic and AKT is antiapoptotic at all tested concentrations of cisplatin, both in the presence and absence of JNK inhibitor SP600125.

Intercellular contacts regulate the cell fate in a variety of ways. Elucidation of the influence of cell−cell contacts on the activity of cell fate-regulating signaling is essential in predicting the cell response to changing conditions. Different influence of cell−cell contacts on c-Jun and JNK activation was shown in our previous work.^16^ Here, by simulating two different cellular states, adherent and unattached to substratum (free-floating), we compared the effect of JNK inhibition on the viability of cells exposed to cisplatin. We found that cells incubated under agitation for 72 h, as described in the Methods, had the same JNK role dependence on cisplatin concentration as cells adherent to the substrate (Supplement Figure S3).

**Figure 8:**
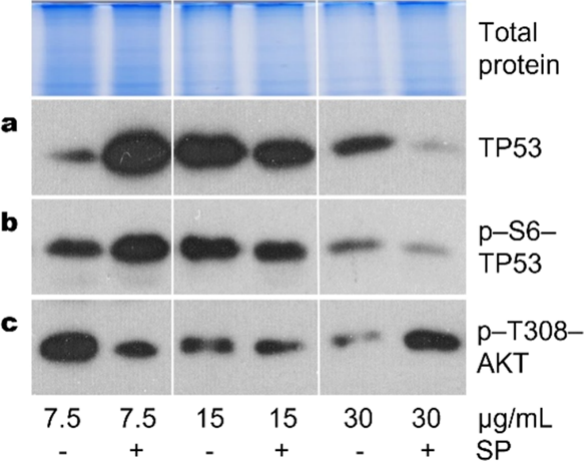
Opposite changes in TP53 and AKT phosphorylation following SP600125 treatment of A549 cells exposed to different concentrations of cisplatin. (a) Expression of TP53 is increased in response to the combination of SP + 7.5 μg/mL cisplatin, in contrast to the combination of SP + 30 μg/mL cisplatin. (b) Phosphorylation of TP53 (serine-6) is increased in response to the combination of SP + 7.5 μg/mL cisplatin, in contrast to the combination of SP + 30 μg/mL cisplatin. (c) Phosphorylation of AKT (threonine-308) is decreased in response to the combination of SP + 7.5 μg/mL cisplatin, in contrast to the combination of SP + 30 μg/mL cisplatin. Representative Western blots are shown. Total protein Coomassie-stained polyacrylamide gels serve as a loading control. SP—JNK inhibitor SP600125 (20 μM); 20 h of treatment.

Based on the above data on the expression, activation, and role of TP53 and AKT in the regulation of cell survival, we next sought to evaluate the possible involvement of the identified molecules in the regulation of cell fate after exposure to JNK inhibitors at different cisplatin concentrations. Our studies suggest that TP53 and AKT may be proteins that mediate different roles of JNK inhibition in A549 cells. Following cisplatin treatment (20 h or longer), transcription factor TP53 protein level as well as TP53 and AKT phosphorylation after JNK inhibition were cisplatin concentration-dependent; JNK inhibition increased TP53 level and phosphorylation in A549 cells at 7.5 μg/mL cisplatin but decreased AKT phosphorylation. Conversely, at higher concentrations of cisplatin (15 and 30 μg/mL), TP53 level and its phosphorylation decreased after SP600125 exposure, while phospho-AKT increased (Figure 8).

In summary, our studies have shown that JNK plays a cell-protective role at low concentrations and a pro-apoptotic or neutral role at higher concentrations of the chemotherapeutic drug cisplatin. Tumor suppressor TP53 and pro-survival kinase AKT could be regarded as potential intracellular targets that possibly mediate opposite cell fate outcomes following JNK inhibitor SP600125 exposure at different cisplatin concentrations in the A549 cell model.

## Discussion

Studies on reducing toxicity and increasing drug potency are the main directions in the search for effective ways to defeat cancer. Usually, chemotherapy-induced toxicities lead to dose reduction or treatment delay. Targeting signal transduction pathways is the most effective way to improve cancer treatment addressing both issues at the same time.^18^

The combination of conventional anticancer drugs with signal-molecule-targeted inhibitors is one of the most promising treatment strategies to overcome resistance. Here, by combining the chemotherapeutic drug cisplatin with JNK signaling pathway inhibitors, we investigated the role of JNK in lung cancer chemoresistance and cell death.

A wide range of studies, with a variety of examples using numerous different cancer models, unequivocally point to JNK as a target for cancer therapy. Currently, designing effective and specific JNK inhibitors is an active area of cancer treatment. JNK pathway plays an important role in cisplatin drug resistance, too. JNK activation was reported upon genotoxic stresses and in response to anticancer treatment including chemotherapy. Cisplatin, a platinum-containing drug, binds and cross-links DNA. In response to cisplatin-induced DNA damage, multiple signaling pathways are activated, the balance of which determines cell fate. Generated reactive oxygen species (ROS) are also potent activators of JNK and other signaling pathways. The status of ROS, the extent of DNA damage, and various downstream factors are responsible for cell fates in response to the cytotoxic effects of DNA-damaging therapies.

Although cisplatin is the mainstay of cancer chemotherapy for many types of tumors, including lung cancer, the optimal dose of cisplatin to be used for cancer treatment is currently being widely discussed. Choosing between a few cycles of high (acute) drug doses (HD) and weekly repeated low (subtoxic) doses (LD) is common for oncologists in cisplatin chemotherapy.^19^ Dose-dependent adverse effects and systemic development of chemoresistance are known. Repeated treatment leads to the selection and emergence of resistant cancer cell populations.

In the past few years, primarily because of HD toxicity, LD weekly chemotherapy became particularly used, although, in general, it was less effective, as concluded from the meta-analysis of 59 clinical trials of head and neck tumor treatment.^20^ Studies show that two doses, a low subtoxic dose (LD; 30 μM) and a 10-fold higher acute dose (HD; 300 μM) of cisplatin, result in clearly different transcriptional responses in colorectal cancer cells in vitro. Alteration of ABC transporters and activation of autophagy was observed at low cisplatin doses, whereas cells exposed to high doses showed a marked increase in intracellular ROS levels, changes in chromatin, and activation of developmental signals.^21^ Studies show that cisplatin resistance is caused by many factors, and the proposed mechanisms of resistance are multiple. Because of the lack of research on the global changes in biological processes caused by cisplatin, cisplatin-regulated signaling pathways are prospectively explored through various -omics technologies, including metabolomics, epigenetics, and single-cell transcriptomics, to find the weak spots and mechanisms of cisplatin resistance.^22^

Thus, identification of differential cellular responses to low or acute doses of cisplatin could be used to guide the selection and usage of appropriate adjuvant(s) to enhance the efficacy of LD- or HD-based treatment. Understanding and applying the obtained results may be valuable in the future to increase the therapeutic efficacy of this widely employed potent anticancer drug.

It is believed that in response to cisplatin activation of the JNK signaling pathway is one of the main factors that determines the fate of the cell. JNK is characterized by functional diversity. In response to various stimuli, spatially and compositionally distinct multiprotein complexes (tissue- and cell-type-dependent) that integrate and transduce signals are assembled. However, in the end, the final response depends on the activity of several signaling pathways that interact with each other, as well as on the type of a cell, or its state.^11^ The major pathways involved in NSCLC are MAPK, PI3K/AKT, and JAK/STAT.^23^

JNKs are proposed as attractive therapeutic targets for various cancers due to their critical tumor-promoting roles. JNK inhibition proved to be effective in exhibiting various anticancer effects, such as blockade of primary tumor growth, impediment of the tumor-initiating potential of cancer stem cells, and inhibition of metastatic disease progression. However, although various types of ATP-competitive and substrate-competitive JNK inhibitors have been developed, their use as anticancer drugs has been limited due to the physiological and tumor-suppressive functions of JNK. At the same time, identification and targeting of the scaffold-JNK and JNK-substrate interfaces, the specific protein−protein interactions within oncogenic JNK signaling complexes, discriminating between the distinct functions of JNK, was proposed as effective and clinically promising therapeutic options and perspective JNK targeting strategy. The composition of JNK signaling complexes is proposed as a promising future research task, perhaps, in the context of tissue dependency.^24^

Currently, JNK inhibitors are not used in clinical therapy due to the necessity of JNK for normal cell functioning and its role in tumor cell apoptosis. These aspects should be kept in mind when developing and researching new generation high selectivity JNK inhibitors.

Being a member of the mitogen-activated protein kinases family (MAPK), the JNK signaling pathway plays a major role in deciding between cell survival and death. Transcription factors c-Jun, ATF2, and TP53 are substrates of activated JNK. The functional diversity of JNK is sometimes characterized by its opposing functions. One of the possible explanations for the different JNK functioning is the pattern and duration of its activation (immediate-early or late, transient or sustained), which is proven to be crucial for the decision to undergo apoptosis. Long-lasting JNK activation is accompanied by sustained upregulation of AP-1 (activator protein-1) and FASL, triggering the death receptor pathway which is thought to induce a sustained subthreshold apoptotic signal, which in turn may fully activate the apoptosis executive machinery over time.

Even though JNK1 and JNK2 isoforms seem to have opposite effects in regulating TP53, which is known as the essential player in response to DNA damage, the JNK pathway is recognized as an upstream regulator of TP53 activity in response to genotoxic chemotherapeutic compounds. In addition to the induction of DNA damage, recent data show that cisplatin promotes the generation of reactive oxygen species (ROS), which play a critical role in cisplatin’s dose-limiting toxicity. Increased ROS production leads to the activation of JNK and concomitantly of TP53 by releasing it from MDM2. Low-dose cisplatin is unable to enhance ROS levels, hence, the degree of ROS accumulation dictates the choice of the downstream pathway.^11^

The tumor suppressor transcription factor TP53 is responsible for diversifying gene expression patterns and can regulate specific cell functions in response to a variety of cellular stresses. The differential regulation of TP53 targets may be dependent on differences in the TP53 phosphorylation profile as a result of different stresses. MAPKs have been shown to post-translationally modify TP53. Different JNK-mediated phosphorylations of TP53 have been reported. For example, direct binding of JNK to TP53 was shown to target TP53 for ubiquitin-mediated degradation,^25^ whereas JNK-mediated TP53 phosphorylation resulted in TP53 accumulation.^26^

Distinct temporal patterns of TP53 expression are described in the literature. They include undamped oscillations, single-grade pulses, and monotonically increased accumulation in response to various stimuli. It depends on the cell line, extent of DNA damage, intrinsic repair rate of DNA lesions, etc. There is evidence that TP53 levels were oscillatory at low stimulatory doses and increased gradually with higher doses of chemotherapeutic drugs.^27,28^ What we demonstrated here is that similar TP53 dynamics were generated in response to the different concentrations of cisplatin (Figure 6) yet lead to different cell fate outcomes. Therefore, TP53 dynamics alone was insufficient to specify the cause of distinct cell fates in response to different concentrations of cisplatin.

The question was raised about why then cells respond differently when TP53 dynamics is similar.

It is known that in response to various stresses and treatments, concomitant with TP53 activation, other parallel signaling pathways are activated/upregulated and interact with each other, including the mitogen-activated protein kinases ERK, JNK, and p38, as well as the protein kinase AKT. Both the crosstalk between these signaling molecules and TP53, as well as TP53 dynamics itself, and integration of these signaling responses may be responsible for the expression of downstream TP53 targets, therefore diversifying cell fate outcomes.^29,30^ The crosstalk between AKT, a well-known pro-survival protein kinase, and JNK kinases was described in various systems.^31^ Moreover, the PI3K/AKT pathway is required in the DNA damage-induced cancer cell death via crosstalk with TP53.^32^

Here, like TP53, activation of AKT was dose- and time-dependent. Selective inhibition of the AKT functioning and activation of TP53 (expression and phosphorylation) led to a decrease in cell survival, indicating their involvement in A549 cell fate determination after cisplatin and cisplatin + SP treatments at all tested concentrations of cisplatin (Figures 6 and 7). The levels of protein expression and phosphorylation in cisplatin + SP-treated A549 cells correlated with their role in cell death regulation. TP53 and AKT were proposed as proteins mediating the different roles of JNK in A549 cells exposed to different cisplatin concentrations. Therefore, transcription factor TP53 and survival kinase AKT could be possible targets in cisplatin-based cancer therapy, combined with inhibition of the JNK pathway.

At this stage of research, we can emphasize that JNK inhibition at low (sublethal) concentrations of cisplatin is particularly effective in killing non-small-cell lung cancer A549 cells. By studying the changes in signaling molecules after exposure to the combination of cisplatin and SP600125, we found that TP53 and AKT may be effectors in the modulation of JNK function in cisplatin-treated cells. The combination of low cisplatin concentrations and JNK inhibition could help effectively remove cancer cells before resistance develops as well as avoid severe side effects associated with cisplatin toxicity.

## Conclusions

The JNK signaling pathway may be a promising target for anticancer therapy in order to improve the efficacy of targeted and conventional chemotherapies. This study suggests that chemotherapy dosing as well as timing of AKT and TP53 signaling may be critical in designing successful regimens of combination treatment. Inhibition of the JNK pathway may be a potential way to increase the sensitivity of lung cancer cells to the chemotherapeutic agent cisplatin. However, the mechanism of action of JNK is highly complex and context-dependent, thus requiring further investigations.

## Data Availability

The data is presented in the article and Supporting material.

## Supporting Information Available

“Cisplatin concentration effect” in serum-free, PI3K/AKT-inhibited and floating A549 cells (PDF)

## Notes

**Ethical approval**: The usage of human lung cancer cell lines established from surgical material was approved by Lithuanian Regional Bioethics Committee (approval no. 158200-18/5-1024-537).

## Notes

**Consent for publication**: All authors approve this submission for publication.

## Abbreviations

JNK: cJun N-terminal kinase; stress-regulated protein kinase of MAPK superfamily;
Cisplatin: cis-diamminedichloroplatinum(II); a chemotherapeutic drug;
NSCLC: non-small-cell lung cancer;
TP53: tumor suppressor p53, a transcription factor;
SP600125: an anthrapyrazolone inhibitor of JNK;
AKT: pro-survival protein kinase, involved in cellular signaling.
