# Supplementary material for: The Role and Efficacy of JNK Inhibition in Inducing Lung Cancer Cell Death Depend on the Concentration of Cisplatin
Source: ACS Omega. 2024 Jun 18;9(26):28311–22. doi: 10.1021/acsomega.4c01950 (PMC11223245; doi:10.1021/acsomega.4c01950)
Supplement: Supplementary file 1 — ao4c01950_si_001.pdf [file ao4c01950_si_001.pdf]

The role and efficacy of JNK inhibition in inducing lung cancer cell death depend on the concentration of cisplatin

Aurimas Stulpinas [1] *,

Monika Tenkutytė [1] ,

Aušra Imbrasaitė [1] ,

Audronė V. Kalvelytė [1] *

[1] Institute of Biochemistry, Life Sciences Center, Vilnius University, Vilnius, Lithuania.

* aurimas.stulpinas@gmc.vu.lt; Saulėtekio av. 7, LT-10257, Lithuania

* audrone.kalvelyte@bchi.vu.lt;_Saulėtekio av. 7, LT-10257, Lithuania

Supporting information

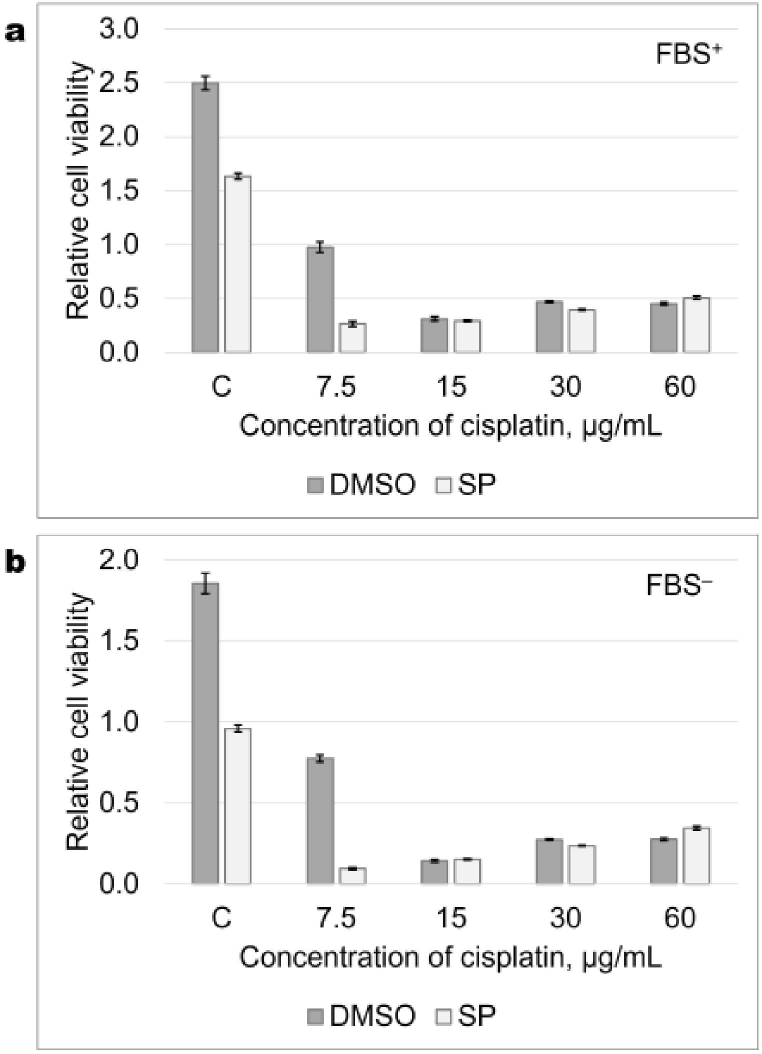

Figure S1. The role of cisplatin concentration in the JNK inhibitory effect is independent of serum/growth factors present in the cell culture medium. (a) SP reduces the viability of A549 cells exposed to cisplatin (7.5 µg/mL) in the presence of FBS (72 hours, MTT assay). (b) SP reduces the viability of A549 cells exposed to cisplatin (7.5 µg/mL) in the absence of FBS (72 hours, MTT assay). SP – JNK inhibitor SP600125 (20 µM), DMSO – vehicle control, FBS – fetal bovine serum (10 %).

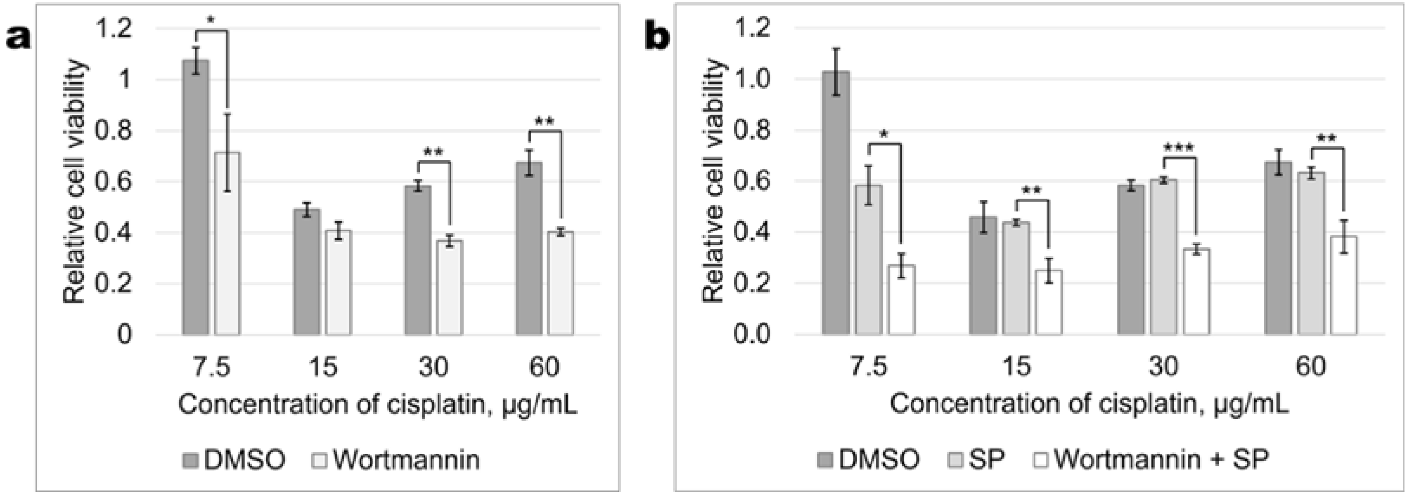

Figure S2. Inhibition of PI3K/AKT pathway augments cell death of cisplatin-treated A549 cells. (a) Addition of Wortmannin reduces the viability of cisplatin-treated cells. (b) Addition of Wortmannin reduces the viability of cisplatin + SP treated cells. Various concentrations of cisplatin were used. Representative test results (all measurements were performed in quadruplicate) from three experiments are presented. $p^{*}<0.05$, $p^{**}<0.005$, $p^{***}<0.0005$, N=4. SP – JNK inhibitor SP600125 (20 µM), DMSO – vehicle control, Wortmannin – PI3K inhibitor (2 µM).

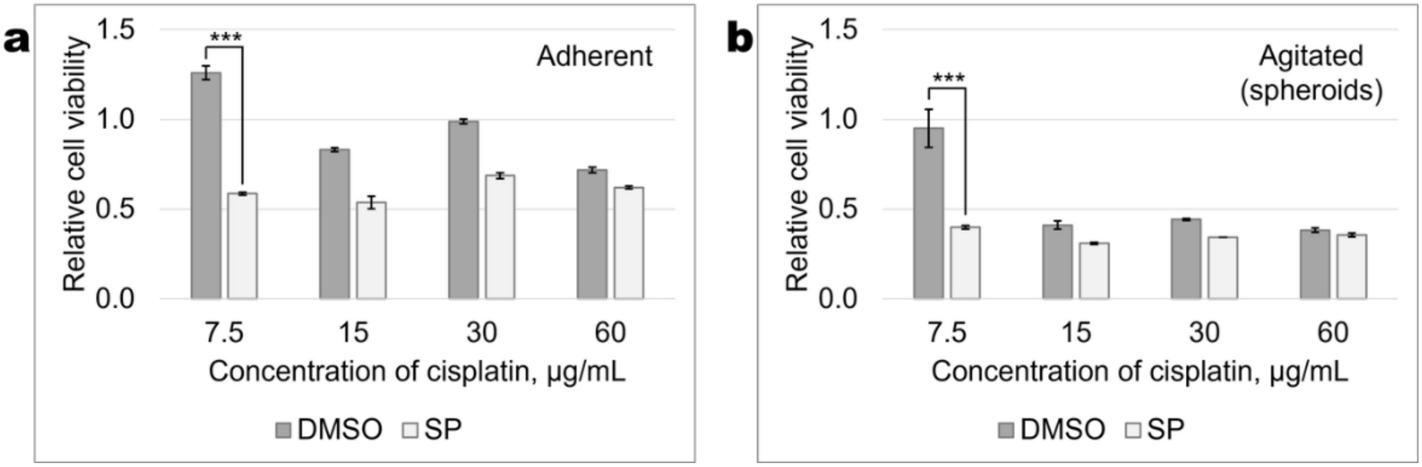

Figure S3. The role of cisplatin concentration in the JNK inhibitory effect is not related to extracellular contacts. (a) A significant decrease in viability of SP + cisplatin (7.5 µg/mL) treated A549 cells is observed in adherent cells (two-dimensional cell culture; 72 hours, resazurin reduction method). (b) A similar significant decrease in viability of SP + cisplatin (7.5 µg/mL) treated A549 cells is observed in agitated spheroids (three-dimensional cell culture; 72 hours, resazurin reduction method). In these experiments, a CO_2-independent cell culture medium was used (see Methods). Representative test results (all measurements were performed in quadruplicate) from more than five experiments are presented. $p^{***}<0.0005$, N=4. SP – JNK inhibitor SP600125 (20 µM), DMSO – vehicle control.
